# Beyond the Introduction: The Next Chapter for *JBMR Plus*

**DOI:** 10.1002/jbm4.10565

**Published:** 2021-10-21

**Authors:** Deborah J. Veis

**Affiliations:** ^1^ Division of Bone and Mineral Diseases, Departments of Medicine and Pathology Washington University School of Medicine St Louis MO USA; ^2^ Department of Pathology and Immunology Washington University School of Medicine St Louis MO USA; ^3^ Musculoskeletal Research Center Washington University School of Medicine St Louis MO USA; ^4^ Shriners Hospitals for Children St Louis MO USA

I am honored to take over the reins at *JBMR Plus* as its second Editor in Chief. Its founding Editor, Peter Ebeling, has done a remarkable job establishing the journal in a very crowded publishing environment, selecting outstanding content and overseeing an exponential growth in views and citations. Now that the journal is off the ground, it is my job to steer it even higher and further by adding more value for our authors and readers. Although I cannot say that becoming an Editor in Chief has been a specific career goal for me, I suppose it is in my blood. My father, Arthur Veis, served in this role at *Connective Tissue Research* for 25 years. I have always enjoyed helping others polish their manuscripts and grants, probably even more than starting my own writing projects “from a blank page.” My last 6 years as an Associate Editor at *JBMR* has exposed me to many new topics and authors in our field and challenged me to provide constructive criticisms and make difficult decisions. Taking this editorial experience together with my own as an author of primary research and literature reviews, I have firsthand appreciation for journal publications, as well as its current challenges, and I am excited about the opportunity to impact the landscape via my own leadership of *JBMR Plus*.

First and foremost, I see *JBMR Plus* as a key asset for the ASBMR and the field of bone and mineral research at large. Although *JBMR* will remain the society's flagship journal, *JBMR Plus* serves as a different resource for our community. *JBMR* can publish only a minority of submitted manuscripts. Among those that are not accepted, many still deserve publication. In some cases, although research has been rigorous, the breadth or depth of the findings may not be felt sufficient for *JBMR*, but the results are sound and the authors are ready to share these results—to support a grant submission, to provide a trainee with a first‐author publication, to describe a smaller story from a departing group member. *JBMR Plus* meets the need of expanded ASBMR publication space for these shorter, more focused but complete manuscripts. Furthermore, as we recognize the importance of reproducibility in biomedical research, publication of replication studies is essential to the research enterprise, and *JBMR Plus* will be a home for validation studies or studies that reproduce previous, non‐validated, or still controversial findings. Technical reports, such as those describing ways to utilize cutting‐edge technologies when working with hard and matrix‐rich tissues such as bone, will be welcome, as they can accelerate research in our field, providing critical resources to the bone and mineral community. On the clinical side, we welcome study protocols and case reports, two publication types of practical utility to the bone and mineral research community.

In addition to increasing the number of manuscripts cascading from *JBMR*, *JBMR Plus* needs to garner more direct submissions. To attract manuscripts, *JBMR Plus* will support authors by providing an easy and transparent process for submission and clear communication throughout. I intend to reach out individually to authors of manuscripts transferred from *JBMR* that appear appropriate for *JBMR Plus*, outlining a potential path to publication and encouraging inquiries about specifics from authors. By engaging in dialog with an editor at this stage, authors can avoid wasting effort on revisions that are unlikely to impact the ultimate decision and can rather focus efforts where they will be most useful. A similar process will be used for direct submissions. The editors will be very clear about the priority of suggestions made by reviewers for subsequent revisions, and inquiries will be welcomed before submission of a full‐scale revision. Although upfront guarantees on publication cannot be made, clear and open communication can help avoid time‐consuming and costly experiments or analyses that are unlikely to affect the outcome, preserving precious resources for their most effective use.

Another way in which a journal can support its research community is by providing career development opportunities. Along with the deputy and associate editors who will join me in leading this effort, I will work to find creative ways to engage a broad range of researchers at multiple career stages in the publications process. We need an engaged editorial board who will evaluate manuscripts in a fashion that is aligned with the mission of the journal. Our ideal article will not have innumerable figure panels and will be more manageable to review, thus representing a good place to learn the art of a good, constructive review. To this end, we plan to produce webinars about best practices for reviewers and assemble a junior editorial board of senior trainees who will be trained and then utilized as regular reviewers alongside more experienced scientists. Young investigators will also be invited to provide short reviews and perspectives about new techniques, hot topics, and meeting reports. Early and substantive participation in journal publications will provide tomorrow's leaders with valuable experience and insights, cracking open what is often seen as a black box along the path of career progression.

In addition to learning how to communicate science, publication of one's work is a way to gain visibility and stature in the field. As a fully online and open access journal, *JBMR Plus* can promote our authors, alongside their work. Therefore, we plan to highlight more of our authors, particularly junior investigators, giving them a platform to share their interests and accomplishments. Manuscripts may be accompanied by short videos explaining a new technique or concept or introducing a newly opened lab. Clinical studies may be paired with video vignettes of related cases, presented by trainees involved in the work or with related interests, providing increased visibility and recognition for the presenter and a valuable learning resource for other trainees. We will also make greater use of social media such as Twitter to engage more readers with our content. As we move forward, I look forward to hearing more ideas to be creative with the array of formats that can be employed for communication of bone and mineral research.

The primary purpose of the journal is to disseminate results from studies—clinical, translational, and basic. The ASBMR Publications Committee recently partnered with the leadership at *JBMR* to update its reporting guidelines to support the highest degree of rigor and transparency, and *JBMR Plus* will now take advantage of this important work and adopt the same high standards. While implementation of these guidelines requires effort from both authors and editors, the end result is a manuscript that is accessible and transparent, effectively communicating the data within. Although *JBMR Plus* and *JBMR* may have unique content profiles, as sister journals of the ASBMR, it makes sense to leverage shared efforts as much as possible.

In closing, I am excited to take on this new challenge, to serve investigators in the dynamic field of bone and mineral research, in all its forms—in the clinic and the lab and everywhere in between. In this endeavor, I will be joined by two Deputy Editors, Drs. Matthew Drake and Hanna Taipaleenmäki. Drs. Peggy Cawthon and Emma Clark have graciously agreed to stay on as Associate Editors, and they will be complemented by Drs. Noriaki Ono and Gabriela Loots. I am especially looking forward to working with this talented team of editors to advance *JBMR Plus* as a valuable resource for both authors and readers, to engage an editorial board of emerging leaders, and to help train the next generation of investigators in scientific publishing.

### Peer Review

The peer review history for this article is available at https://publons.com/publon/10.1002/jbm4.10565.

